# An R Package for Bayesian Analysis of Multi-environment and Multi-trait Multi-environment Data for Genome-Based Prediction

**DOI:** 10.1534/g3.119.400126

**Published:** 2019-02-28

**Authors:** Osval A. Montesinos-López, Abelardo Montesinos-López, Francisco Javier Luna-Vázquez, Fernando H. Toledo, Paulino Pérez-Rodríguez, Morten Lillemo, José Crossa

**Affiliations:** * Facultad de Telemática, Universidad de Colima, Colima, Colima, 28040, México; † Departamento de Matemáticas, Centro Universitario de Ciencias Exactas e Ingenierías, (CUCEI), Universidad de Guadalajara, Guadalajara, Jalisco, 44430, México; ‡ Biometrics and Statistics Unit, International Maize and Wheat Improvement Center, (CIMMYT), Apdo. Postal 6-641, Ciudad de México, 06600, México; § Colegio de Postgraduados, CP 56230, Montecillos, Edo. de México, México; ** Department of Plant Sciences, Norwegian University of Life Sciences, IHA/CIGENE, P.O. Box 5003, NO-1432 Ås, Norway

**Keywords:** multi-environment, multi-trait, genome-based prediction and selection, R-software, multivariate analysis, GenPred, Shared data resources, Genomic Prediction

## Abstract

Evidence that genomic selection (GS) is a technology that is revolutionizing plant breeding continues to grow. However, it is very well documented that its success strongly depends on statistical models, which are used by GS to perform predictions of candidate genotypes that were not phenotyped. Because there is no universally better model for prediction and models for each type of response variable are needed (continuous, binary, ordinal, count, etc.), an active area of research aims to develop statistical models for the prediction of univariate and multivariate traits in GS. However, most of the models developed so far are for univariate and continuous (Gaussian) traits. Therefore, to overcome the lack of multivariate statistical models for genome-based prediction by improving the original version of the BMTME, we propose an improved Bayesian multi-trait and multi-environment (BMTME) R package for analyzing breeding data with multiple traits and multiple environments. We also introduce Bayesian multi-output regressor stacking (BMORS) functions that are considerably efficient in terms of computational resources. The package allows parameter estimation and evaluates the prediction performance of multi-trait and multi-environment data in a reliable, efficient and user-friendly way. We illustrate the use of the BMTME with real toy datasets to show all the facilities that the software offers the user. However, for large datasets, the BME() and BMTME() functions of the BMTME R package are very intense in terms of computing time; on the other hand, less intensive computing is required with BMORS functions BMORS() and BMORS_Env() that are also included in the BMTME package.

Genomic selection (GS) is a methodology used in plant breeding that was proposed by Meuwissen *et al.* (2001). It is a type of marker-assisted selection that consists of genotyping and phenotyping a training sample (reference population); with the help of statistical models, predictions of genomic estimated breeding values (GEBV) or phenotypic values of the testing sample (validation population) are obtained for which only genome-wide dense genetic marker data were available. GS does not depend on prior knowledge about a few, large-effect genes or QTL, since all markers are used simultaneously in the training of the statistical models. GS was first used in animal breeding (Hayes and Goddard 2010), but nowadays is being implemented in many crops, for example, maize (Crossa *et al.*, 2014), cassava (de Oliveira *et al.*, 2012), wheat (Rutkoski *et al.*, 2011), sugar beet (Würschum *et al.*, 2013), tomato (Yamamoto *et al.*, 2016), rice (Spindel *et al.*, 2015), apple (Kumar *et al.*, 2012), pea (Burstin *et al.*, 2015), cranberry (Covarrubias-Pazaran *et al.*, 2018) and many others.

In recent years, an active area of research has begun to develop and improve existing statistical models for genomic selection (GS) due to the fact that successful GS implementation is strongly related to the accuracy of the predictions performed by statistical models. However, because there are no universally superior machines for prediction, many models have been proposed. For example, most of the proposed models are univariate and few are multivariate. Most of the univariate models are appropriate for continuous and Gaussian phenotypes, but there are several appropriate models for binary, ordinal and count traits. Some examples of implementations of models for non-Gaussian, non-continuous traits are unordered categorical (Heuer *et al.*, 2016), binomial (Technow and Melchinger 2013) and ordinal categorical (Montesinos-López *et al.*, 2015a,b). While multivariate models are used almost routinely nowadays, for the joint analysis of multiple-traits (*e.g.*, Jia and Jannink 2012) as well as multiple-environments (*e.g.*, Burgueño *et al.*, 2012) and even multiple populations (*e.g.*, Olson *et al.*, 2012), there are few multivariate practical software programs for continuous and Gaussian phenotypes and there are scarcely any models and software for other types of response variables. To the best of our knowledge, almost none of the currently reported models consider mixed phenotypes including continuous, binary, ordinal, count, etc. traits. For this reason, it is clear that to increase the power of GS technology, it is of paramount importance to develop more models and improve the existing ones.

Multi-trait models in GS have been applied by many scientists. For example, Calus and Veerkamp (2011), Jia and Jannink (2012), Jiang *et al.* (2015), He *et al.* (2016), Schulthess *et al.* (2017), and Covarrubias-Pazaran *et al.* (2018) reported that multi-trait analysis outperforms uni-trait analysis in terms of prediction accuracy and that the larger the correlation between traits, the larger the benefit of multi-trait analysis. The Multi-Trait Model (MTM) of de los Campos and Grüneberg (2016) is a mixed multi-trait Gaussian model under the Bayesian framework that uses a Gibbs sampler for inferences. Furthermore, Bayesian multi-output regressor stacking (BMORS) is a Bayesian version of the multi-trait regressor stacking method proposed by Spyromitros-Xioufis *et al.* (2012; 2016). The training of BMORS has two stages: (1) a single univariate model is implemented using the GBLUP model, and (2) the resulting predictions are directly included by BMORS in an additional training stage. Thus, the concept of BMORS is that a second-stage model will correct the predictions of the first-stage model [using the predictions of the first-stage univariate GBLUP model (Spyromitros-Xioufis *et al.*, 2012; 2016)].

Montesinos-Lopez *et al.* (2016) were the first to develop a comprehensive theory for a Bayesian multi-trait multi-environment (BMTME) model for genome-based prediction. An improved version of BMTME allows general covariance matrices by using the matrix normal distribution that facilitates easy derivation of all full conditional distributions and permits a more efficient model in terms of time of implementation Montesinos-López *et al.* (2018a,b,c). In general, the matrix normal distribution model considerably improved in terms of implementation time over the time required by the original BMTME. Also, the Gibbs sampler for implementing the new BMTME model can be found in Montesinos-López *et al.* (2018a), and the priors of the model are given in detail in Montesinos-López *et al.* (2018b). Montesinos-López *et al.* (2018a) provide the appropriate notations used for the matrix-variate normal distribution that is a generalization of the multivariate normal distributions to matrices. This plays a key role in building the BMTME model. The original software used by Montesinos-Lopez *et al.* (2016) to fit the BMTME was the first attempt to implement the multi-trait multi-environment theory when analyzing real data; however, the lack of the necessary optimization algorithms for efficiently applying the software made the original BMTME difficult to apply to real data.

It is also important to point out that even though the existing R statistical software for Bayesian analysis like ’stan’ (https://mc-stan.org/) and ‘JAGS’ (https://en.wikipedia.org/wiki/Just_another_Gibbs_sampler) are very flexible for implementing Bayesian analysis, they are not user-friendly because the user needs a certain level of programming skills to correctly implement them (Stan Development Team 2018; Plummer 2018). These two software programs (stan and JAGS) also require more computational resources for their implementation since they are built not with conjugate priors. It is documented that multivariate analysis improves parameter estimation (Schulthess *et al.*, 2017). For this reason, we agree with Castro *et al.* (2013) and Huang *et al.* (2015), who stated that multi-trait analysis is a powerful tool for clarifying the relationship and the effect of each studied variable and for building more efficient prediction models.

Due to the background of plant breeders, not only are new models needed, but the existing ones need to be improved. We also need reliable, efficient, user-friendly software in which breeders can implement the existing GS models. One popular R package in the context of genomic selection for continuous and ordinal data are the BGLR package of Pérez and de los Campos (2014) that was built under the Bayesian framework and is very flexible because it allows the use of a genomic relationship matrix (derived from marker or pedigree), and also allows implementing various methods like BayesA, BayesB, Bayes Lasso, Bayes Ridge and GBLUP and can deal with moderate datasets; however, it only allows the implementation of univaritate models. Therefore, to contribute to this requirement, we developed a Bayesian multi-trait and multi-environment (BMTME) R software that allows the implementation of multi-trait and multi-environment data for performing parameter estimates and evaluating the prediction performance of multiple traits that are studied in many environments. This BMTME package is different from existing ones [sommer (Covarrubias-Pazaran 2016), BGGE (Granato *et al.*, 2018), ASREML (Gilmour *et al.*, 1995) and MCMCglmm (Hadfield *et al.*, 2010)] because it takes into account the genetic correlation between traits and between environments. The main difference of BMTME with sommer and ASREML is that our package was built under a Bayesian framework, while sommer and ASREML were based on a classical approach using restricted maximum likelihood. The difference between BGGE and our model is that our model is not only for multi-environment data but rather for multi-environment and multi-trait data simultaneously. On the other hand, the MCMCglmm package only allows a general covariance matrix for traits but not for environments, like the proposed BMTME package; however, it is important to point out that the MCMCglmm package allows modeling not only continuous responses but also binary, ordinal and counts.

The main objective of this research was to illustrate the application of the new BMTME with two real toy datasets; with these we show how to use the functions available in the BMTME package for implementing multi-environment (BME function), multi-trait and multi-environment data (BMTME function), as well as the Bayesian multi-output regressor stacking functions BMORS () and BMORS_ENV (). These two functions are very different to what the existing software [sommer (Covarrubias-Pazaran 2016), BGGE (Granato *et al.*, 2018), ASREML (Gilmour *et al.*, 1995) and MCMCglmm (Hadfield *et al.*, 2010)] implements, since the theory behind this function is that of stacking methods. Stacking methods consist of training multiple learning algorithms for the same dataset and then combining the predictions to obtain the final predictions. In this study we used the initial BMTME of Montesinos-Lopez *et al.* (2016) but improved it by using the matrix variate normal distribution (Montesinos-López *et al.*, 2018c) and the appropriate priors given by Montesinos-López *et al.* (2018a) and Montesinos-López *et al.* (2018b).

## Methods

### Statistical models

#### Multiple-environment Genomic Best Linear Unbiased Predictor (GBLUP) model:

Since genotype × environment interaction is of paramount importance in plant breeding, the following univariate linear mixed model is often used for each trait:

$$y_{ij}=E_{i}+g_{j}+gE_{ij}+e_{ij}$$ (1)

where $y_{ij}$ represents the response of the jth line in the ith environment (i=1,2,…,I, j=1,2,…,J). $E_{i}\;$represents the effect of the ith environment, $g_{j}$ represents the random genomic effect of the jth line, with $g={\left(g_{1},\ldots ,g_{J}\right)}^{T}∼N\left(0,\sigma_{1}^{2}\;G_{g}\right),$ $\sigma_{1}^{2}$ is a genomic variance, $G_{g}$ is of order J×J and represents the genomic relationship matrix (GRM) and is calculated (VanRaden 2008) as $G_{g}=\frac{WW^{T}}{p}$, where p denotes the number of markers and W is the matrix of markers of order J×p. The $G_{g}\;$matrix is constructed using the observed similarity at the genomic level between lines, rather than the expected similarity based on pedigree. Further, $gE_{ij}$ is the random interaction term between the genomic effect of the jth line and the ith environment with $\mathrm{gE}={\left(gE_{11},\ldots ,gE_{IJ}\right)}^{T}∼N\left(0,\sigma_{2}^{2}\;I_{I}⊗G\right)$, where $\sigma_{2}^{2}$ is an interaction variance, and $e_{ij}$ is a random residual associated with the jth line in the ith environment distributed as $N(0,\sigma^{2})$ where $\sigma^{2}$ is the residual variance.

#### Bayesian multiple-trait multiple-environment (BMTME) model:

The current BMTME model was implemented by Montesinos-López *et al.* (2018a,b,c). For a complete understanding of its description, first we provide the notations used for the matrix-variate normal distribution that plays a key role in building the BMTME model. Matrix-variate normal distribution is a generalization of the multivariate normal distribution to matrices. The (*n*×*p*) random matrix, M, has a matrix-variate normal distribution denoted as $M∼NM_{n\times p}\left(H,\Omega ,\Sigma \right)$, if and only if, the (*np*×1) random vector vec(M) is distributed as multivariate normal as $N_{np}\left(\text{vec}(H),\Sigma ⊗\Omega \right)$; therefore, $NM_{n\times p}$ denotes the (n×p) dimensional matrix-variate normal distribution, H is a (*n* × *p*) location matrix, Σ is a (p×p) first covariance matrix, and Ω is a (n×n) second covariance matrix (Srivastava and Khatri 1979). vec(.) and ⊗ are the standard vector operator and Kronecker product, respectively. Unlike in a multivariate normal model where the data are concatenated into a single vector of length *np*, in a matrix-variate normal model, the data (M) are in an *n*×*p* matrix where each column is a trait (Montesinos-López *et al.*, 2018a). Therefore, the proposed BMTME model is defined as follows:

$$Y=\mathrm{X\beta}+Z_{1}b_{1}+Z_{2}b_{2}+E$$ (2)

where Y is of order n×L, with L the number of traits and n=J×I, where J denotes the number of lines and I the number of environments, X is of order n×I, β is of order I×L, since $\beta =\{\beta_{il}\}$ for i=1,..,I and l=1,..,L, $Z_{1}$ is of order n×J, $b_{1}$ is of order J×L and contains the genotype × trait interaction term since $b_{1}=\{gt_{jl}\}$ where $gt_{jl}$ is the effect of the genotype × trait interaction term for l=1,..,J and for j=1,..,L.$\;Z_{2}$ is of order n×IJ, $b_{2}$ is of order IJ×L and contains the genotype × environment × trait interaction, since $b_{2}=\{gEt_{jil}\}$, where $gEt_{jil}$ is the effect of genotype × environment × trait interaction for j=1,..,J, for i=1,..,I and for l=1,..,L. Vector $b_{1}\;$is distributed under a matrix-variate normal distribution as $NM_{J\times L}\left(0,G_{g},\Sigma_{t}\right),$ where $G_{g}$ is of order J×J and represents the Genomic Relationship Matrix (GRM) and is calculated using the VanRaden (2008) method as $G_{g}=\frac{WW^{T}}{p}$, where p denotes the number of markers and W the matrix of markers of order J×p; and $\Sigma_{t}$ is the unstructured genetic (co)variance matrix of traits of order L×L, $b_{2}∼NM_{JI\times L}\left(0,\Sigma_{E}⊗G_{g},\Sigma_{t}\right)$, where $\Sigma_{E}\;$is an unstructured (co)variance matrix of order I×I and E is the matrix of residuals of order n×L with $E∼NM_{n\times L}\left(0,I_{n},R_{e}\right)$, where $R_{e}$ is the unstructured residual (co)variance matrix of traits of order L×L, and $G_{g}$ is the genomic relationship matrix described above (Montesinos-López *et al.*, 2018a). The BMTME model resulting from equation (2) was implemented by Montesinos-Lopez *et al.* (2016).

Next, we used the modified version of the Gibbs sampler of the original BMTME model proposed by Montesinos-Lopez *et al.* (2016) that was implemented in Montesinos-López *et al.* (2018a). It is important to point out that model (2) takes into account the genotype × environment terms in the ($Z_{2}b_{2})$ term and, for comparison purposes, we also ran the model in equation (2) but without the ($Z_{2}b_{2})$ term to study the effect on prediction performance with and without the genotype × environment term. The Gibbs sampler for implementing the BMTME model is found in Montesinos-López *et al.* (2018a), and the priors of this model are given in detail in Montesinos-López *et al.* (2018b). The concept of the matrix variate normal distribution is given in Montesinos-López *et al.* (2018c).

#### Bayesian multi-output regressor stacking (BMORS):

The proposed BMORS is a Bayesian version of the multi-trait regressor stacking method proposed by Spyromitros-Xioufis *et al.* (2012; 2016). The training of BMORS consists of two stages. In the first stage, L single univariate models are implemented using the GBLUP model given in equation (1), but instead of using the resulting predictions directly as the final output, the BMORS includes an additional training stage where a second set of L meta-models are implemented for each of the L traits under study. Each meta-model is implemented with the following model:

$$y_{ij}=\beta_{1}{\hat{Z}}_{1ij}+\beta_{2}{\hat{Z}}_{2ij}+\ldots +\beta_{L}{\hat{Z}}_{Lij}+e_{ij}$$ (3)

where the covariates ${\hat{Z}}_{1ij},{\hat{Z}}_{2ij},\ldots ,{\hat{Z}}_{Lij}$ represent the scaled predictions of each trait obtained with the GBLUP model in the first-stage analysis, and $\beta_{1},\ldots ,\beta_{L}$ are the beta coefficients for each covariate. The scaling of each prediction was performed by subtracting its mean ($\mu_{lij}$) and dividing by its corresponding standard deviation ($\sigma_{lij}$), that is, ${\hat{Z}}_{lij}$=$({\hat{y}}_{lij}-\mu_{lij})\sigma_{lij}^{-1}$, for each l=1,…,L. Therefore, the BMORS model contains as predictor information the scaled predictions of its response variables yielded by the first-stage models. In other words, the BMORS model is based on the idea that a second-stage model is able to correct the predictions of a first-stage model using information about the predictions of other first-stage models (Spyromitros-Xioufis *et al.*, 2012; 2016).

### Real toy datasets

#### Mada dataset:

This dataset was obtained from the study by Ben Hassen *et al.* (2018). The dataset is composed of a sample of 188 wheat lines evaluated for six traits. Each of the lines was evaluated in one environment. The lines were genotyped and 32,066 single nucleotide polymorphisms (SNPs) were obtained with a heterozygosity rate < 5% and a minor allele frequency (MAF) > 5%. A subset of the data were included in the package that includes 30 lines, and we named this dataset Mada. For more details, see the study by Ben Hassen *et al.* (2018). Raw markers are not included, and we provide the genomic relationship matrix (GRM) calculated according to the method of VanRaden (2008).

#### Maize dataset:

This dataset was obtained from the study by Montesinos-Lopez *et al.* (2016). It consists of a sample of 309 maize lines evaluated for three traits: anthesis-silking interval (ASI), plant height (PH), and grain yield (GY). Each trait was evaluated in three optimal environments (Env1, Env2 and Env3). The lines were genotyped, 681,257 single nucleotide polymorphisms (SNPs) were obtained, and markers with more than 20% missing values were removed. After that, markers were imputed using observed allelic frequencies, and markers with MAF < 0.05 were removed, so that at the end of the quality control and imputation, 158,281 SNPs were still available for further analyses. To load this dataset in the package, we used only 30 lines, and we named this dataset Maize. For more details, see the study by Montesinos-Lopez *et al.* (2016).

### Evaluation of prediction performance

We implemented cross-validation (CV) to evaluate the prediction performance. Two types of CV were implemented: K-fold cross-validation and random cross-validation.

#### K-fold cross-validation:

Under this CV, the dataset was partitioned into K subsamples of equal size; each time K-1 of them were used for training (TRN) and the remaining one for testing (TST). In this CV, one observation cannot appear in more than one fold. In the design, some lines can be evaluated in some, but not all, target environments, which mimics a prediction problem faced by breeders in incomplete field trials. This CV strategy is exactly the same as the strategy denoted as CV2 that was proposed and implemented by Jarquín *et al.* (2017), where a certain portion of test lines (genotypes) in a certain portion of test environments is predicted, since some test lines that were evaluated in some test environments are assumed to be missing in others.

#### Random cross-validation:

This CV strategy randomly splits the dataset into training (TRN) and testing data (TST). For each such split, the model is fitted to the TRN data, and predictive accuracy is assessed using the TST data. Since we used sampling with replacement, one observation may appear in more than one partition. The implemented CV mimics a prediction problem faced by breeders in incomplete field trials, where some lines may be evaluated in some, but not all, target environments. Since N=J×I denotes the total number of records per each available trait, then to select lines in the TST dataset, we fixed the percentage of data to be used for TST [PTesting]. Then PTesting×N (lines) were chosen at random, and subsequently for each of these lines, one environment was randomly picked from I environments. The cells selected through this algorithm were allocated to the TST dataset, while the cells (ij) that were not selected were assigned to the TRN dataset. Lines were sampled with replacement if J<PTesting×N, and without replacement otherwise (López-Cruz *et al.*, 2015). The metrics used to measure the prediction accuracy under both CV strategies were Pearson’s correlation and the mean arctan absolute percentage error (MAAPE), which has the advantage that no zero estimates are produced when the response variable contains many zeros. They were calculated from each trait-environment combination for each of the testing sets and the average of all random partitions (folds) is reported as a measure of prediction performance.

### Data availability

The data used in this study are included in the BMTME package, so once that package is installed, the datasets can be loaded into the R environment.

### Installation of the BMTME package

The aim of this section is to illustrate the use of the R BMTME package for analyzing multi-environment and multi-trait and multi-environment data from plant breeding programs. The BMTME package was built following the paper by Montesinos-Lopez *et al.* (2016) and implemented in the R statistical software (R Core Team 2018).

The development version of the BMTME package can be installed directly from the GitHub repository (https://github.com/frahik/BMTME). In order to install the package, it is necessary to install the appropriate compilers; the installation process and the required tools depend heavily on the operating system. For example, in Windows it is necessary to install Rtools (https://cran.r-project.org/bin/windows/Rtools/), and in modern versions of macOS, it is necessary to install XCode from App Store or the development tools for R from CRAN (https://cran.r-project.org/bin/macosx/tools/). In the case of Linux, it is necessary to install the C++ compilers included in your distribution, for example, g++ from GNU (https://www.gnu.org). Once the tools have been installed, use the following command to install the package within your R session:

install.packages(′devtools′)
devtools::install_github(′frahik/BMTME′)

You can also find the package in the CRAN repository, and you can use the following command (see below) to install a version of the package from CRAN. This will avoid the need to install some dependencies manually and install the Rtools software using the following command:

install.packages(′BMTME′)

The R package BMTME is available at the following link: https://cran.r-project.org/web/packages/BMTME/index.html.

## Results

The results are given in three main sections. The first section illustrates the use of the BME function for implementing multi-environment analysis, while the second and the third sections illustrate the use of the BMTME and BMORs functions for implementing multi-trait and multi-environment analyses.

### The BME Function

This example illustrates how to fit a model when there is only one environment and several dependent variables. First, we load the library:

library(BMTME)

Then we load the Mada dataset:

data(“WheatMadaToy”)

Then we define the model to be adjusted; since the dataset only includes an environment where several dependent variables were evaluated, the BME model is used. To implement it, first we need to order the dataset as follows:

phenoMada <- (phenoMada[order(phenoMada$GID),])
rownames(phenoMada)=1:nrow(phenoMada)
head(phenoMada)
GID PH FL FE NS SY NP
1 9 29.7776 -8.8882 -4.93900 1.04100 169.06 28.8025
2 11 3.2210 -7.1111 -0.36940 -3.88940 -107.19 58.2516
3 12 6.1670 -9.5337 -12.43680 2.58250 -160.54 17.1278
4 15 6.8117 4.6377 11.78860 -0.03378 235.70 -19.6571
5 20 -14.4480 3.2525 6.40780 -14.23460 131.87 42.2962
6 21 -13.2185 3.8902 0.09722 5.35680 164.06 36.8239

This is a very important step in the analysis, because if the dataset is not ordered correctly, this may cause conflicts and produce incorrect estimations. Also, with the head() function we printed the phenotypic dataset, where the required format of the dataset requires a first column with the identifiers of the lines and then the names of all the traits. It is important to respect this format to be able to successfully implement the multi-environment (trait) datasets.

Then, the design matrix for the genetic effects should be generated, as shown below.

LG <- cholesky(genoMada)
ZG <- model.matrix(∼0 + as.factor(phenoMada$GID))
Z.G <- ZG %*% LG

Then, we can extract the phenotypic responses that were converted to matrix object as shown in the following command:

Y <- as.matrix(phenoMada[, -c(1)])

Finally, the model was adjusted, and 30,000 iterations were used to adjust the model.

fm <- BME(Y = Y, Z1 = Z.G, nIter = 30000, burnIn = 20000, thin = 2, bs = 50)

It is important to point out that bs is the block size for sampling from posterior distributions; we suggest using a value of at least 50 but less than 1000.

Next we used the names() function to identify all the available outputs of the fitted model.

names(fm)
[1] “Y” “nIter” “burnIn” “thin” “dfe”
[6] “Se” “yHat” “SD.yHat” “beta” “SD.beta”
[11] “b1” “SD.b1” “vare” “SD.vare” “varTrait”
[16] “SD.varTrait” “NAvalues”

Here we extracted the observed values ($Y), the predicted values ($yHat), the parameters provided for the model fit ($nIter, $burnIn, $thin, etc.), the estimates of the beta coefficients, random effects of lines and the genetic and residual covariances ($beta, $SD.beta, $b1, $SD.b1, $varTrait, $vare, etc.). Next we show how to extract the predicted values:

head(fm$yHat)
PH FL FE NS SY NP
[1,] 13.4602 -4.6073 2.2247 -5.2839 -161.7740 28.9334
[2,] 4.6792 -2.7261 2.3857 -3.3243 -89.1425 29.2848
[3,] 3.0779 -2.0297 0.1369 -3.2161 -91.4642 11.7453
[4,] 5.2408 1.6600 5.6141 1.6136 60.2424 -23.3492
[5,] -5.1899 1.0555 1.8783 -3.0015 49.6383 11.7256
[6,] -9.8591 1.2399 0.5886 3.4539 88.8181 26.9665

We also extracted the genetic covariance between traits, as shown below:

COV_TraitGenetic <- fm$varTrait
COV_TraitGenetic
PH FL FE NS SY NP
[1,] 64.4266 -4.2148 6.3730 -0.0049 -103.8781 -114.4456
[2,] -4.2148 5.9607 3.2257 -0.1355 175.7770 -21.0909
[3,] 6.3730 3.2257 23.8617 1.3721 -133.4962 -43.6292
[4,] -0.0049 -0.1355 1.3721 46.2903 370.1770 -59.0136
[5,] -103.8781 175.7770 -133.4962 370.1770 27963.1634 604.6203
[6,] -114.4456 -21.0909 -43.6292 -59.0136 604.6203 872.3525

To convert this covariance matrix into a correlation matrix, we suggest using the following command:

COR_TraitGenetic <- cov2cor(COV_TraitGenetic)
COR_TraitGenetic
PH FL FE NS SY NP
[1,] 1.000000e+00 -0.215077873 0.16254014 -8.972603e-05 -0.07739239 -0.4827480
[2,] -2.150779e-01 1.000000000 0.27047346 -8.157286e-03 0.43054670 -0.2924829
[3,] 1.625401e-01 0.270473457 1.00000000 4.128479e-02 -0.16342747 -0.3023991
[4,] -8.972603e-05 -0.008157286 0.04128479 1.000000e+00 0.32536534 -0.2936710
[5,] -7.739239e-02 0.430546701 -0.16342747 3.253653e-01 1.00000000 0.1224175
[6,] -4.827480e-01 -0.292482897 -0.30239914 -2.936710e-01 0.12241752 1.0000000

Here there are no high correlations (*i.e.*, greater than 0.5). In a similar way, we obtained the residual covariance matrix:

COV_ResGenetic <- fm$vare
COV_ResGenetic
PH FL FE NS SY NP
[1,] 65.2173 -2.7253 -12.3196 20.9669 880.3571 -71.9332
[2,] -2.7253 11.6654 10.6847 -7.8144 182.0011 -10.3907
[3,] -12.3196 10.6847 40.1432 -20.4498 -436.0834 -31.4401
[4,] 20.9669 -7.8144 -20.4498 54.0458 375.0032 -70.7700
[5,] 880.3571 182.0011 -436.0834 375.0032 60960.1184 2058.4876
[6,] -71.9332 -10.3907 -31.4401 -70.7700 2058.4876 670.1587

**Figure 1:**
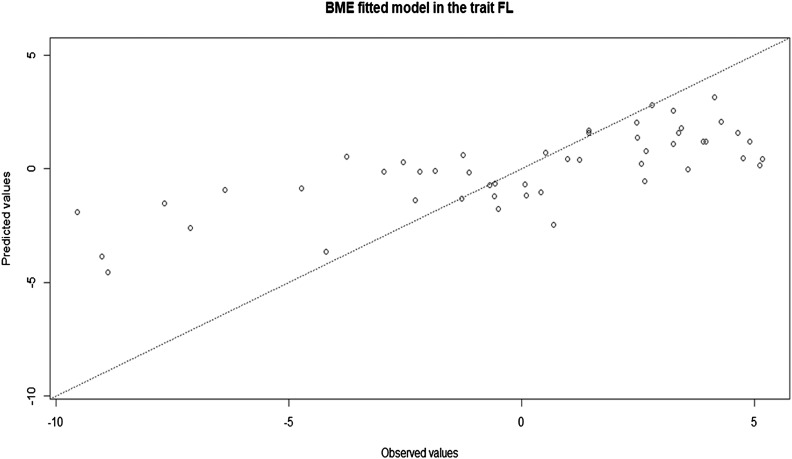
Observed and predicted values for trait FL resulting from fitting the model with the BME function to the Mada dataset.

For demonstration purposes, we will only extract the first 6 predictions for the 6 evaluated traits. We also plotted the observed values against the predicted values for each trait, as follows (see Figure 1):

plot(fm, trait = ′FL′)

Since the code provided above is only appropriate for parameter estimation, now we provide the code required to evaluate the prediction accuracy using the BME() function. For this reason, first we built the random CV strategy with 10 random partitions, each with TRN = 80% and TST = 20%, using the following code:

pheno <- data.frame(GID = phenoMada[, 1], Response = phenoMada[, 2])
CrossV <- CV.RandomPart(pheno, NPartitions = 10, PTesting = 0.2, set_seed = 123)
Finally, we implemented the CV strategy as:
pm <- BME(Y = Y, Z1 = Z.G, nIter = 1250, burnIn = 500, thin = 2, bs = 50, testingSet = CrossV)

In the summary we show the average predictions of the 10 random partitions implemented under average Pearson's correlation (APC) and MAAPE:

summary(pm)
Environment Trait Pearson SE_Pearson MAAPE SE_MAAPE
1 FE 0.3895 0.0847 0.7264 0.0313
2 FL 0.1758 0.0767 0.7751 0.0248
3 NP 0.3920 0.1192 0.7070 0.0347
4 NS 0.4281 0.0879 0.7604 0.0364
5 PH 0.5612 0.0767 0.7285 0.0542
6 SY 0.0242 0.0623 0.7436 0.0255

**Figure 2:**
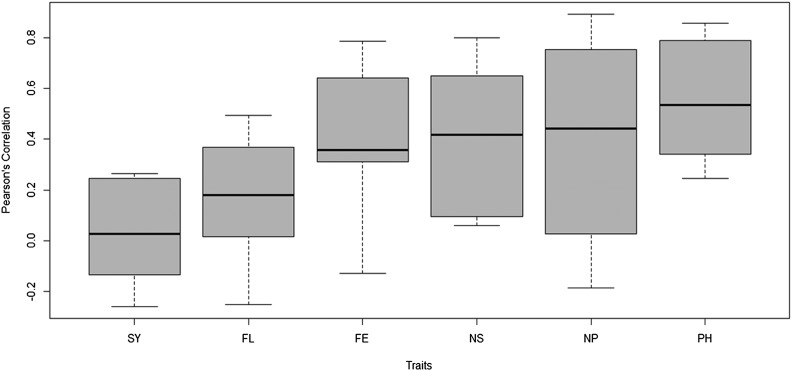
Boxplot of Pearson’s correlation of the testing sets for each trait of the Mada data-set.

Here we see that the best prediction in terms of APC was found in trait PH (0.5612), while the worst was in trait SY (0.0242). However, in terms of MAAPE, the best prediction was observed in trait NP (0.7070), while the worst was found in trait FL (0.7751). With the boxplot(pm) function, we created a plot summarizing the predictions in terms of Pearson’s correlation, but if users want this plot in MAAPE terms, they need to use the following code: boxplot(pm, select=“MAAPE”) (Figure 2).

boxplot(pm)

It is important to point out that the BME function can be used with only 1 testing set that can be defined by the user, as shown in the following example:

CrossV1 <-sample(nrow(Y),15)
pm <- BME(Y = Y, Z1 = Z.G, nIter = 1250, burnIn = 500, thin = 2, bs = 50,testingSet = CrossV1)

Next we summarize the prediction accuracy:

summary(pm)
Environment Trait Pearson SE_Pearson MAAPE SE_MAAPE
1 NA FE 0.1181 NaN 0.7765 NaN
2 NA FL -0.1222 NaN 0.8207 NaN
3 NA NP 0.6937 NaN 0.6813 NaN
4 NA NS 0.4636 NaN 0.7098 NaN
5 NA PH 0.7362 NaN 0.7949 NaN
6 NA SY -0.0079 NaN 0.8251 NaN

Since only one training set and one testing set were used, the standard errors for both metrics appear with NaN, given that it is not possible to calculate the standard error because only one testing set is available.

### The Bayesian Multi-Trait and Multi-Environment (BMTME) function

This example illustrates how to fit a model with multiple traits and multiple environments. To do this, use the Maize dataset; first, load the data using the following function:

data(“MaizeToy”)

Next, order the dataset, rename the rows of the phenotypic dataset and print the first six observations of the data in order to see the structure required of the data, which consists of a first column that includes the lines, a second column that includes the environments and third, fourth and fifth columns that correspond to the traits under study.

phenoMaizeToy<-(phenoMaizeToy[order(phenoMaizeToy$Env,phenoMaizeToy$Line),])
rownames(phenoMaizeToy)=1:nrow(phenoMaizeToy)
head(phenoMaizeToy)
Line Env Yield ASI PH
1 CKDHL0008 EBU 6.88 2.7 226
2 CKDHL0039 EBU 6.85 1.3 239
3 CKDHL0042 EBU 6.37 2.3 238
4 CKDHL0050 EBU 4.98 3.1 239
5 CKDHL0060 EBU 7.07 1.4 242
6 CKDHL0063 EBU 8.62 2.3 250
7 CKDHL0069 EBU 5.16 1.0 248
8 CKDHL0072 EBU 5.77 1.7 227

This step is very important for avoiding an incorrect estimation process. Then the design matrices for the line effects, the environment and the genotype×environment interaction are generated:

LG <- cholesky(genoMaizeToy)
ZG <- model.matrix(∼0 + as.factor(phenoMaizeToy$Line))
Z.G <- ZG %*% LG
Z.E <- model.matrix(∼0 + as.factor(phenoMaizeToy$Env))
ZEG <- model.matrix(∼0 + as.factor(phenoMaizeToy$Line):as.factor(phenoMaizeToy$Env))
G2 <- kronecker(diag(length(unique(phenoMaizeToy$Env))), data.matrix(genoMaizeToy))
LG2 <- cholesky(G2)
Z.EG <- ZEG %*% LG2
Y <- as.matrix(phenoMaizeToy[, -c(1, 2)])

Finally, the following command is used to fit the model:

fm <- BMTME(Y = Y, X = Z.E, Z1 = Z.G, Z2 = Z.EG, nIter =15000, burnIn =10000, thin = 2,bs = 50)

We used the names() function to see all the things that can be extracted after fitting a model with the BMTME function.

names(fm)
[1] “Y” “nIter” “burnIn” “thin” “dfe” “Se” “yHat” [8] “SD.yHat” “beta” “SD.beta” “b1” “b2” “vare” “SD.vare”
[15] “varEnv” “SD.varEnv” “varTrait” “SD.varTrait” “NAvalues”

We can extract the predicted and observed values, the random effects of lines, of lines×trait, lines×environment×trait, as well as the genetic covariances between traits and environments, and the residual covariance between traits. To extract the matrix of covariances between traits, we used the following:

COV_TraitGenetic <- fm$varTrait
COV_TraitGenetic
Yield ASI PH
[1,] 0.0956 -0.0027 0.9642
[2,] -0.0027 0.0654 0.1893
[3,] 0.9642 0.1893 23.0647

To convert this covariance matrix between traits into a correlation matrix, we used the following command:

COR_TraitGenetic <- cov2cor(COV_TraitGenetic)
COR_TraitGenetic
Yield ASI PH
[1,] 1.00000000 -0.03414648 0.6493282
[2,] -0.03414648 1.00000000 0.1541302
[3,] 0.64932822 0.15413023 1.0000000

Next we show how to extract the matrix of genetic covariance between the environments.

COV_EnvGenetic <- fm$varEnv
COV_EnvGenetic
EBU KAK KTI
[1,] 308.6270 258.9984 285.3723
[2,] 258.9984 237.9283 264.3881
[3,] 285.3723 264.3881 324.4536

Finally, we show how to extract the residual covariance matrix between traits.

COV_ResGenetic <- fm$vare
COV_ResGenetic
Yield ASI PH
[1,] 0.5282 -0.0955 3.3701
[2,] -0.0955 0.4837 -1.5896
[3,] 3.3701 -1.5896 106.5525

**Figure 3:**
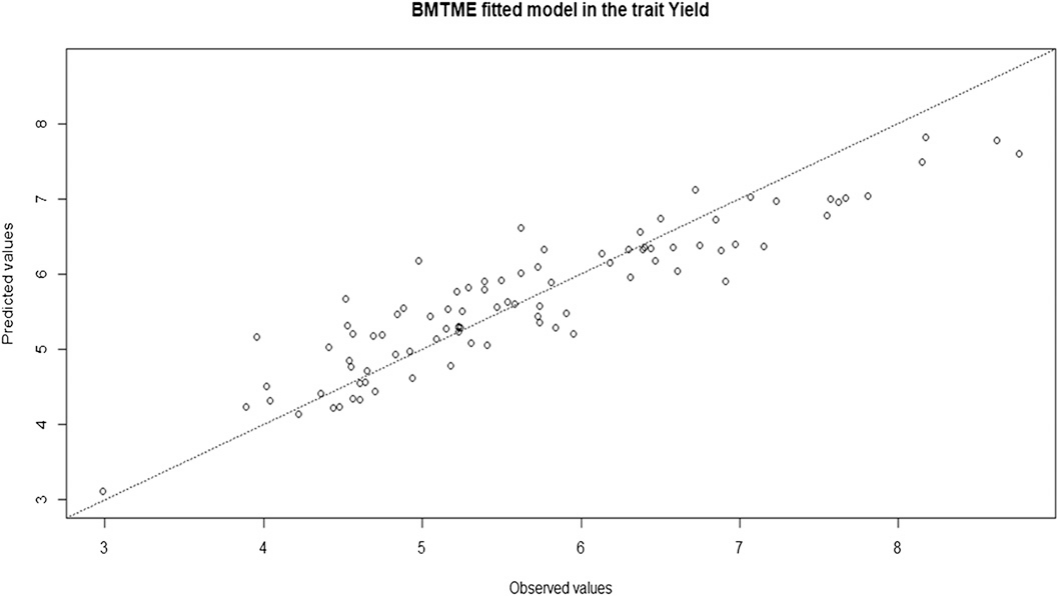
Observed and predicted values for trait Yield resulting from fitting the model with the BMTME function to the Maize dataset.

The observed and predicted values of each trait can be plotted using the following plot(), function (Figure 3):

plot(fm, trait=′Yield′)

We provide the corresponding code for the fivefold CV strategy and its implementation with the BMTME function:

pheno <- data.frame(GID = phenoMaizeToy[, 1], Env = phenoMaizeToy[, 2], Response = phenoMaizeToy[, 3])
CrossV <- CV.KFold(pheno, DataSetID = ′GID′, K = 5, set_seed = 123)
pm <- BMTME(Y = Y, X = Z.E, Z1 = Z.G, Z2 = Z.EG, nIter = 1250, burnIn = 500, thin = 2,bs = 50, testingSet = CrossV)

With the summary we obtained the average predictions in terms of Pearson’s correlation and MAAPE for each trait × environment combination.

summary(pm)
Environment Trait Pearson SE_Pearson MAAPE SE_MAAPE
1 EBU ASI 0.4136 0.1997 0.3128 0.0369
2 EBU PH 0.2141 0.1227 0.0553 0.0054
3 EBU Yield 0.1064 0.2358 0.1554 0.0153
4 KAK ASI 0.1711 0.3141 0.5771 0.0375
5 KAK PH 0.5918 0.1907 0.0462 0.0097
6 KAK Yield 0.6233 0.1642 0.1161 0.0162
7 KTI ASI 0.0064 0.1436 0.3421 0.0300
8 KTI PH 0.2599 0.2212 0.0631 0.0103
9 KTI Yield 0.6077 0.1241 0.1390 0.0116

**Figure 4:**
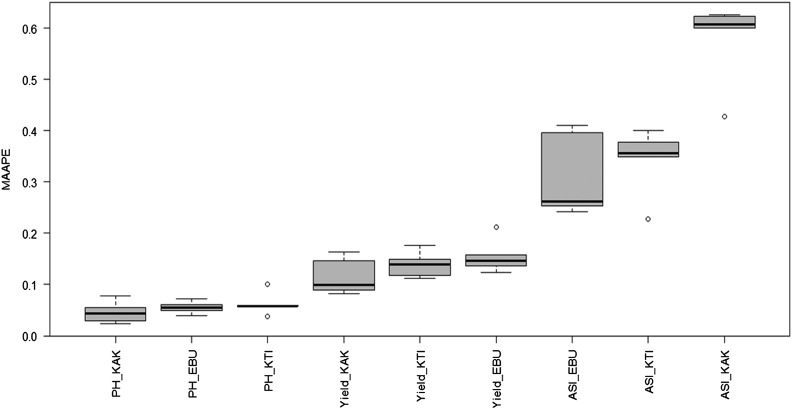
MAAPE of the testing sets for each trait-environment combination of the Maize dataset using the BMTME function.

The summary information is given with the following code using the boxplot(pm, select=”MAAPE”, las = 2), where we added the parameter las = 2, to show the labels vertically and be able to distinguish the complete names (Figure 4).

boxplot(pm, select=”MAAPE”, las = 2)

### The BMORS Function

Since the BMORS function is only appropriate for evaluating prediction performance (but not for parameter estimation), we provide the required R script for evaluating the prediction performance of multiple trait and multiple environment data with a random CV strategy. Since we use the same dataset that was used to illustrate the BMTME function, we only provide the R code for building the predictor (ETA), the CV strategy and for implementing the BMORS function. To build the linear predictor, we used the following R code:

ETA <- list(Env = list(X = Z.E, model = “BRR”), Gen = list(X = Z.G, model = “BRR”), EnvGen = list(X = Z.EG, model = “BRR”))

Next, we provide the R code for implementing the random CV strategy:

CrossValidation <-CV.RandomPart(pheno, NPartitions = 10, PTesting = 0.2, set_seed = 123)

Finally, the model will be implemented with 15,000 iterations, of which 10,000 will be used as burn-in to fit the model. Below we show the resulting predictive performance using the summary() function. The information = ′complete′ command inside the summary() function shows the prediction performance (in terms of Pearson’s correlation and MAAPE) for all partitions implemented, but we only show the first 20 observations.

pm <- BMORS(Y, ETA = ETA, nIter = 15000, burnIn = 10000, thin = 2, progressBar = TRUE, testingSet = CrossValidation, digits = 4)
head(summary(pm, information = ′complete′), 20)
Environment Trait Partition Pearson MAAPE
1 EBU Yield 1 0.4815 0.1693
2 EBU Yield 2 0.1231 0.1170
3 EBU Yield 3 0.2922 0.2016
4 EBU Yield 4 0.5016 0.2224
5 EBU Yield 5 -0.1674 0.2701
6 EBU Yield 6 0.1033 0.1774
7 EBU Yield 7 0.6086 0.0919
8 EBU Yield 8 0.6689 0.1878
9 EBU Yield 9 0.3322 0.1068
10 EBU Yield 10 0.1161 0.1966
11 EBU ASI 1 0.3506 0.3080
12 EBU ASI 2 0.2944 0.2764
13 EBU ASI 3 0.0694 0.4862
14 EBU ASI 4 0.9157 0.3902
15 EBU ASI 5 0.7654 0.2694
16 EBU ASI 6 0.7956 0.2843
17 EBU ASI 7 0.8630 0.3009
18 EBU ASI 8 -0.1160 0.3421
19 EBU ASI 9 0.1343 0.2799
20 EBU ASI 10 0.8346 0.2041

The summary of the predictions is obtained with the following code:

summary(pm)
Environment Trait Pearson SE_Pearson MAAPE SE_MAAPE
1 EBU Yield 0.3060 0.0835 0.1741 0.0175
2 EBU ASI 0.4907 0.1219 0.3142 0.0245
3 EBU PH 0.3486 0.1612 0.0482 0.0050
4 KAK Yield 0.3075 0.1019 0.0965 0.0079
5 KAK ASI 0.0064 0.1296 0.5733 0.0449
6 KAK PH 0.6010 0.0650 0.0396 0.0037
7 KTI Yield 0.3816 0.0992 0.1639 0.0104
8 KTI ASI 0.0278 0.0973 0.2878 0.0198
9 KTI PH 0.3835 0.0753 0.0591 0.0063

**Figure 5:**
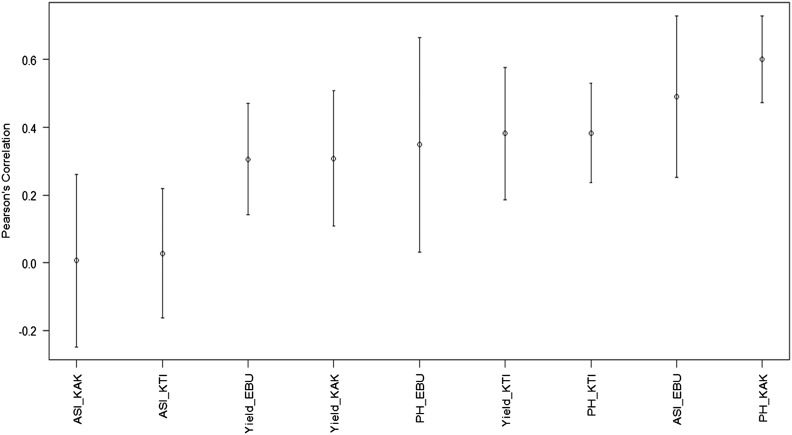
Average Pearson’s correlation of the testing sets for each trait-environment combination of the Maize dataset using the BMORS function.

To create a graph with a summary of the predictions in terms of Pearson’s correlation and in terms of MAAPE, we used the plot() function (Figure 5). Because the names are composed of the evaluated traits and environments, we added the parameter las = 2 to show the labels in a vertical way and to distinguish the complete names of the trait-environment combinations. In addition, we used the par() function and the mar parameter to modify the margins of the graph.

par(mar = c(6,4,2,1))
plot(pm, las = 2)

**Figure 6:**
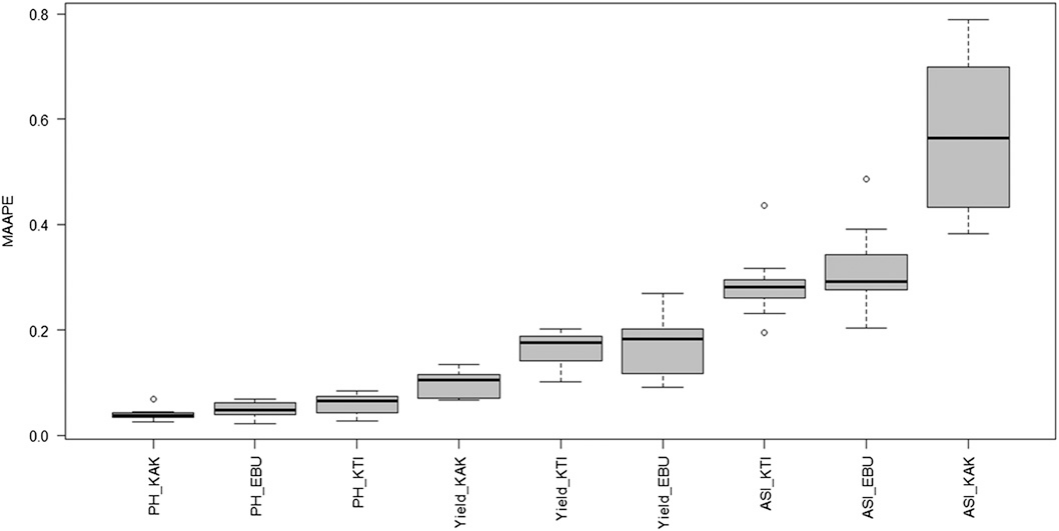
Boxplot of MAAPE of the testing sets for each trait-environment combination of the Maize dataset using the BMORS function.

Figure 5 shows that the lowest average Pearson’s correlation obtained was observed in the ASI_KAK and ASI_KTI trait-environment combinations, while the highest average Pearson’s correlation was obtained in the PH_KAK trait-environment combination. It is possible to create a boxplot with the results of the MAAPE, using the following command (Figure 6):

boxplot(pm, select =”MAAPE”, las = 2)

Figure 6 shows that the lowest MAAPE was for PH_KAK (best prediction), while the highest MAAPE was for the ASI_KAK trait-environment combination (worst prediction).

### The BMORS_ENV Function

This function is useful for predicting whole environments using the remaining environments as training. Next we provide the R code for evaluating the prediction performance of the same maize dataset, but using the KAK environment as training and the KTI and EBU environments as testing. Two important things to point out for using this function are: (a) that we provided not only the matrix of response variables, but also a data.frame that contains, in the first column, the names of the environments followed by information on all response variables, and (b) we did not create a separate file for specifying the training and testing individuals; we only specified in testingEnv which environments are used as testing; the remaining environments are used by default as training, as shown below.

dataset <- phenoMaizeToy[, 2:5]
pm <- BMORS_Env(dataset, testingEnv = **c**(′KTI′, ′EBU′), ETA = ETA, covModel = ′BayesB′, nIter = 15000, burnIn = 10000, thin = 2, progressBar = TRUE, digits = 3)
summary(pm)
Environment Trait Pearson MAAPE
1 KTI Yield 0.4114 0.1344
2 KTI ASI 0.0334 0.4304
3 KTI PH 0.2091 0.0760
4 EBU Yield 0.2775 0.1722
5 EBU ASI -0.0586 0.3825
6 EBU PH 0.0533 0.0933

**Figure 7:**
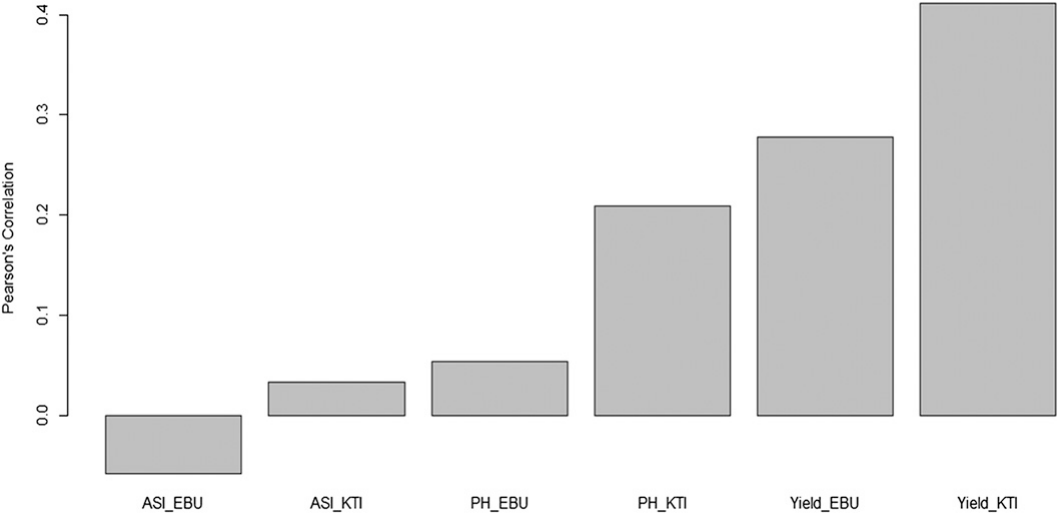
Average Pearson’s correlations of the testing sets for each trait-environment combination of the Maize dataset using the BMORS_Env function.

For this example, we specified that covModel = ′BayesB′, which means that the Bayesian BayesB model will be implemented for the second stage of the model where it is implemented (equation 3). In covModel, in addition to Bayesian Ridge regression (BRR) and BayesB, we can also implement BayesA, BayesC and Bayesian Lasso (BL); however, the BRR model is implemented by default.To create a graph with Pearson’s correlation or the MAAPE index, we used the barplot() function, as shown below (Figure 7):

barplot(pm)

Figure 7 shows that the lowest Pearson’s correlation obtained was in the ASI_EBU trait-environment combination, while the highest Pearson’s correlation was obtained in the Yield_KTI trait-environment combination.

## Discussion

**Figure 8:**
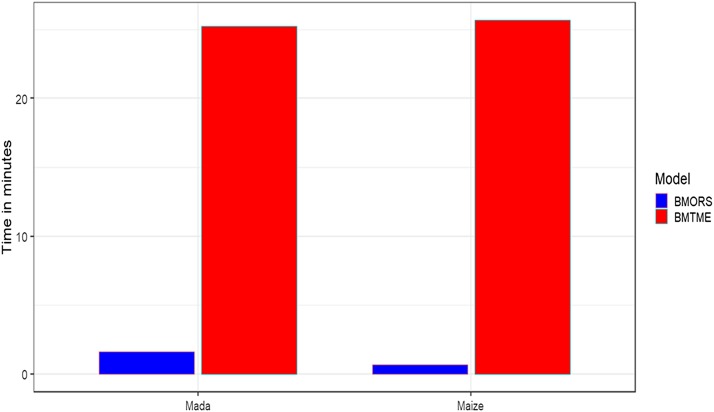
Implementation time in minutes between the BMTME and BMORS models under the Mada and Maize datasets.

As mentioned in the introduction, we propose a Bayesian R package for implementing multi-environment and multi-trait and multi-environment analysis for parameter estimation and for evaluating prediction accuracy. We illustrate the four main functions [BME(), BMTME(), BMORS() and BMORS_Env()] of the BMTME package with real toy datasets starting from the type and preprocessing required to make correct use of each of these datasets for parameter estimation and for evaluating prediction performance. It is important to point out that one advantage of the BME and BMTME functions is that, in addition to being used to evaluate the prediction accuracy, they can also be used for parameter estimation, which allows estimating the random effects (lines, lines×environments for each trait) and variance-covariance matrices of genetic (for traits and environments) and residual (for traits) effects. The BMORS() and BMORS_Env() functions are not useful for obtaining parameter estimates of covariances between traits and environments because they implement univariate analysis at both stages. However, they have two important advantages: (a) they allow implementing even more complex predictors than the one specified in equation (1), which modifies the ETA list used to create the predictor, and (b) the computational resources required to implement it are much less than those needed by the BMTME() function for implementing multi-trait and multi-environment data. This last point is observed in Figure 8 where the implementation time for the Mada and Maize datasets is reported. The figure shows that in the Mada dataset, the BMORS model was more than 15 times faster than the BMTME model (25.246/ 1.621= 15.572), while in the Maize dataset, the BMORS model was more than 37 times faster than the BMTME model (25.668/ 0.692= 37.093); these results were obtained with 10000 iterations of the Gibbs sampler. The BMTME R package provides very synthetic summaries (tables and plots) of the prediction accuracies, which are ready to be interpreted and used to write the results in a manuscript. Additionally, we provide three types of cross-validations useful for breeders that are implemented in this R package, which is very simple to use and implement.

The main disadvantage of the BME() and BMTME() functions of the BMTME R package is that the computational resources required for their implementation are very demanding; fortunately, the parameter estimates involved are stabilized very quickly even with few iterations. The toy examples used in this article are for illustration purposes and to help users follow, as easily as possible, the necessary steps for running the different processes. Comparing them with other software of similar type is not possible, as no similar software for simultaneously fitting multi-trait multi-environment is currently available. For example, the MTM (de los Campos and Grüneberg 2016) is an efficient Bayesian multi-trait software but is not multi-trait and multi-environment. Future research on benchmarking the BME() and BMTME() functions of the BMTME package with other potential software to be developed in terms of computing time for processing large datasets should be performed. However, the BMORS() and BMORS_Env() functions that also belong to the BMTME R package are very efficient in terms of computational resources, which gives the user an alternative option for performing this type of analyses.

It is important to point out that the proposed BMTME package is different from existing multi-trait analysis software such as ASREML (Gilmour *et al.*, 1995), sommer (Covarrubias-Pazaran 2016), BGGE (Granato *et al.*, 2018) and MCMCglmm (Hadfield *et al.*, 2010). In addition to taking into account variance-covariance matrices of traits (genetic and residual), it also takes into account the genetic covariance (correlation) between environments, which is estimated from the data. This can help improve parameter estimates and prediction accuracy when the degree of correlation between traits is moderate or high.

Multi-trait models are preferred over univariate-trait models because they have the following advantages: (a) they produce higher prediction accuracy because they have more information (direct or indirect) and better data connectedness (Colleau *et al.*, 2009); (b) they improve index selection because optimal weight factors can be obtained for the total merit index (Colleau *et al.*, 2009); and (c) they allow obtaining more precise genetic and residual covariances and incorporating them into expected breeding value (EBV) estimates for across-location, across-country or across-region evaluations (Thompson and Meyer 1986; Schaeffer 2001).

Note that the two datasets used for illustrating the main functions of the BMTME R package are datasets with few lines (toy datasets) with the main intention that users interested in using the package can obtain results very quickly and practice using the software. However, the structure of the data are exactly the same as the structure of the data produced in plant breeding programs. The two toy datasets are included in the BMTME package to facilitate its implementation and allow users to practice using the R software.

To conclude, this paper presents the R package BMTME which allows the implementation of multi-trait, multi-trait and multi-environment analysis for estimating parameters (genetic correlation between traits and environments, residual correlation between traits, random effects of lines and lines×environments) and evaluating the prediction accuracies of many traits simultaneously. We illustrate the implementation of the main functions (BME, BMTE and BMORS) of the R package with two toy real datasets that are very common in plant breeding programs. We provide details of the characteristics that each of the datasets must have, and show how to build the CV strategies available in the package, how to prepare the data to implement the main functions of the BMTME package, how to extract the parameter estimates and how to obtain the summary and plots of prediction accuracies resulting from the implemented CV strategy. The computing time of the BME() and BMTE() functions of the BMTME R package for large datasets is significantly more demanding (in terms of time) than for the toy examples used in this study.

## APPENDIX A – R CODE

### R code for example 1.

rm(list=ls())

library(BMTME)

data(“WheatMadaToy”)

ls()

phenoMada <- (phenoMada[order(phenoMada$GID),])

rownames(phenoMada)=1:nrow(phenoMada)

head(phenoMada)

LG <- cholesky(genoMada)

ZG <- model.matrix(∼0 + as.factor(phenoMada$GID))

Z.G <- ZG %*% LG

Y <- as.matrix(phenoMada[, -c(1)])

head(Y)

##############Parameter estimation##############################

fm <- BME(Y = Y, Z1 = Z.G, nIter =15000, burnIn =10000, thin = 2, bs = 50)

names(fm)

head(fm$yHat)

COV_TraitGenetic <- fm$varTrait

COV_TraitGenetic

COR_TraitGenetic <- cov2cor(COV_TraitGenetic)

COR_TraitGenetic

COV_ResGenetic <- fm$vare

COV_ResGenetic

COR_ResGenetic <- cov2cor(COV_ResGenetic)

COR_ResGenetic

head(fm$yHat)

plot(fm,trait=”FL”)

plot(fm,trait=”FE”)

#############Evaluation of prediction performance#################

pheno <- data.frame(GID=phenoMada[, 1],

Response = phenoMada[, 2])

CrossV <- CV.RandomPart(pheno, NPartitions = 10, PTesting = 0.2, set_seed = 123)

pm <- BME(Y = Y, Z1 = Z.G, nIter = 1250, burnIn = 500, thin = 2, bs = 50,

testingSet = CrossV)

summary(pm)

boxplot(pm)

boxplot(pm, select=”MAAPE”)

CrossV1 <-sample(nrow(Y),15)

CrossV1

pm <- BME(Y = Y, Z1 = Z.G, nIter = 1250, burnIn = 500, thin = 2, bs = 50,

testingSet = CrossV1)

summary(pm)

boxplot(pm)

boxplot(pm, select=”MAAPE”)

### R code for example 2.

rm(list=ls())

library(BMTME)

data(“MaizeToy”)

phenoMaizeToy<-(phenoMaizeToy[order(phenoMaizeToy$Env,phenoMaizeToy$Line),])

rownames(phenoMaizeToy)=1:nrow(phenoMaizeToy)

head(phenoMaizeToy,8)

LG <- cholesky(genoMaizeToy)

ZG <- model.matrix(∼0 + as.factor(phenoMaizeToy$Line))

Z.G <- ZG %*% LG

Z.E <- model.matrix(∼0 + as.factor(phenoMaizeToy$Env))

ZEG <- model.matrix(∼0 + as.factor(phenoMaizeToy$Line):as.factor(phenoMaizeToy$Env))

G2 <- kronecker(diag(length(unique(phenoMaizeToy$Env))), data.matrix(genoMaizeToy))

LG2 <- cholesky(G2)

Z.EG <- ZEG %*% LG2

Y <- as.matrix(phenoMaizeToy[, -c(1, 2)])

fm <- BMTME(Y = Y, X = Z.E, Z1 = Z.G, Z2 = Z.EG, nIter =1500, burnIn =1000, thin = 2,bs = 50)

str(fm)

COV_TraitGenetic <- fm$varTrait

COV_TraitGenetic

COR_TraitGenetic <- cov2cor(COV_TraitGenetic)

COR_TraitGenetic

COV_EnvGenetic <- fm$varEnv

COV_EnvGenetic

COR_EnvGenetic <- cov2cor(COV_EnvGenetic)

COR_EnvGenetic

COV_ResGenetic <- fm$vare

COV_ResGenetic

COR_ResGenetic <- cov2cor(COV_ResGenetic)

COR_ResGenetic

plot(fm, trait=′Yield′)

plot(fm,trait=”ASI”)

####Evaluation of prediction performance with BMTME###############

pheno <- data.frame(GID = phenoMaizeToy[, 1], Env = phenoMaizeToy[, 2],

Response = phenoMaizeToy[, 3])

CrossV <- CV.KFold(pheno, DataSetID = ′GID′, K = 5, set_seed = 123)

pm <- BMTME(Y = Y, X = Z.E, Z1 = Z.G, Z2 = Z.EG, nIter = 1250, burnIn = 500, thin = 2,bs = 50, testingSet = CrossV)

summary(pm)

boxplot(pm, select =”MAAPE”, las = 2)

####Evaluation of prediction performance with BMORS####################

ETA <- list(Env = list(X = Z.E, model = “BRR”), Gen = list(X = Z.G, model = ’BRR’), EnvGen = list(X = Z.EG, model = “BRR”))

CrossValidation <-CV.RandomPart(pheno, NPartitions = 10, PTesting = 0.2,set_seed = 123)

pm <- BMORS(Y, ETA = ETA, nIter = 15000, burnIn = 10000, thin = 2, progressBar = TRUE, testingSet = CrossValidation, digits = 4)

head(summary(pm, information = ′complete′), 20)

par(mar=c(6,4,2,1))

plot(pm, las = 2)

boxplot(pm, select =”MAAPE”, las = 2)

###########Evaluation of prediction performance with BMORS_Env################################

dataset <- phenoMaizeToy[, 2:5]

pm1 <- BMORS_Env(dataset, testingEnv = c(’KTI’, ’EBU’), ETA = ETA, covModel = ’BayesB’, nIter = 1500, burnIn = 1000, thin = 2,

progressBar = TRUE, digits = 3)

summary(pm1)

barplot(pm1)

barplot(pm1,select=”MAAPE”)
